# Risk profiles before suicide mortality in patients with bipolar disorder across the lifespan

**DOI:** 10.1192/j.eurpsy.2025.2451

**Published:** 2025-04-21

**Authors:** Yueh-Pin Lin, Wen-Yin Chen, Chun-Hung Pan, Sheng-Siang Su, Shang-Ying Tsai, Chiao-Chicy Chen, Chian-Jue Kuo

**Affiliations:** 1 Taipei City Psychiatric Center, Taipei City Hospital, Taipei, Taiwan; 2 Department of Counseling Psychology, Chinese Culture University, Taipei, Taiwan; 3 School of Medicine, College of Medicine, Fu Jen Catholic University, New Taipei City, Taiwan; 4 Department of Psychology, National Chengchi University, Taipei, Taiwan; 5 Psychiatric Research Center, Taipei Medical University Hospital, Taipei, Taiwan; 6 Department of Psychiatry, School of Medicine, College of Medicine, Taipei Medical University, Taipei, Taiwan; 7 Department of Psychiatry, Mackay Memorial Hospital, Taipei, Taiwan; 8 Department and Graduate Institute of Forensic Medicine, College of Medicine, National Taiwan University

**Keywords:** bipolar disorder, lifespan, nested case–control study, risk profile, suicide mortality

## Abstract

**Background:**

Studies examining age-stratified risk factors for suicide among individuals with bipolar disorder in different stages of life are scant, possibly because of the insufficient number of suicide cases.

**Aim:**

This study investigated suicide mortality rates and risk profiles of suicide mortality stratified by five age groups in individuals with bipolar disorder.

**Methods:**

This study identified patients with a diagnosis of bipolar disorder between January 1, 2000, and December 31, 2021, from Taiwan’s National Health Insurance Research Database. The study population comprised 45,211 inpatients diagnosed with bipolar disorder, with 1,370 suicide cases during the study period. We calculated the standardized mortality ratio (SMR) of the bipolar cohort relative to the general population. In the age-stratified nested case–control study, risk set sampling was performed to match 1 suicide case with 10 living controls by age, sex, and the year of first diagnosis. The age-stratified risk associated with demographic characteristics, psychiatric and physical comorbidities was estimated using multivariable conditional logistic regression.

**Results:**

The highest SMR (47.0) for suicide was observed in individuals with bipolar disorder aged <30 years. SMR decreased with age; patients aged >60 years had an SMR of 9.5. Among those younger than 40 years, a higher percentage of unemployment was noted among suicide cases than among controls. A significantly increased risk of the depressive phase of bipolar disorder was noted shortly before suicide mortality among patients with bipolar disorder in all age groups. Drug-induced and alcohol-induced mental disorders were associated with suicide and were highly prevalent in patients aged <30 years. Other forms of heart disease were identified in patients aged <40 years, and pneumonia was detected in the 50–59 years age group.

**Conclusions:**

These findings aid the development of health-care intervention strategies for preventing suicide among patients with bipolar disorder in various stages of life.

## Introduction

Mortality risk is significantly higher in individuals with bipolar disorder than in individuals in the general population [1–3]. A study in Taiwan [1] reported that the life expectancies at birth for patients with bipolar disorder were all significantly lower than the national norms; specifically, the results revealed 14.71 years and 12.69 years of life lost for men and women with bipolar disorder, respectively. Suicide is a major public health concern globally, with 700,000 suicides occurring annually [4]. Relative to other psychiatric disorders, bipolar disorder is associated with a notably high prevalence of suicide attempts and completed suicides [5, 6]. Globally, the risk of suicide is 15–30 times higher in patients with bipolar disorder than in individuals in the general population [3, 5, 7, 8]. The average onset age of bipolar affective disorder is between 20 and 30 years. The risk of suicide is higher in the first few years after the onset of bipolar disorder; however, the risk could remain higher in patients with bipolar disorder, even among older patients, than in the general population [3, 5].

Studies [2, 6, 9] have revealed several risk factors for suicide in patients with bipolar disorder, including sex, marital status, age, unemployment, previous suicide attempts, a family history of suicide, rapid cycling pattern, comorbid psychosis, genetic predisposition, alcohol or drug use disorder, and a history of childhood abuse. Bipolar disorder entails fluctuating symptoms throughout the lifespan, affecting interpersonal relationships and occupational functioning. Psychological and social conditions with varying stressors at different ages may influence the risk of suicide in patients with bipolar disorder. However, an investigation into how the risk factors for bipolar disorder vary across different age groups is lacking.

The risk factors leading to suicide mortality shortly before suicide mortality are mainly investigated through psychological autopsy studies, in which the circumstances before suicide are reconstructed [10, 11]. Big data databases provide large samples for investigating the risk factors for suicide, offering an opportunity for conducting age-stratified analyses of the risk factors. For examining the age-stratified risk profiles of suicide mortality across the lifespan of patients with bipolar disorder, sufficient suicide cases in each age stratum are required.

In this study, to obtain data on a sufficient number of suicide cases, the data of patients with bipolar disorder were linked with the national mortality database in Taiwan. Thus, this study enrolled a large-scale cohort of patients with bipolar disorder. With an age-stratified nested case–control study design with a clear temporal relationship in bipolar patients with various age groups, this study investigated the risk profiles and risk factors (demographic characteristics, health-care utilization, and comorbidities) of these individuals prior to their death by suicide. The findings aid in the formulation of age-specific suicide prevention measures in this population.

## Methods

### Data sources

Taiwan’s National Health Insurance Research Database (NHIRD) contains the medical claims data of the entire Taiwanese population. Various high-impact epidemiological and clinical studies have utilized this validated database [2, 12, 13]. In the present study, patients with a diagnosis of bipolar disorder (ICD-9-CM codes 296.0–296.16, 296.4–296.81, 296.89, and 296.9; ICD-10-CM codes F30.x and F31.x) at any time between January 1, 2001, and December 31, 2021, were identified from the database (*n* = 45,211). The date of the first diagnosis of bipolar disorder served as the index date. This study was approved by the Taipei City Hospital Research Ethics Committee (Approval No. TCHIRB-10911013), which waived the requirement of informed consent due to the anonymized and retrospective nature of the data.

### Identification of suicide mortality cases

To determine the mortality status of each patient, the data of patients with bipolar disorder were linked to the national mortality database for the period spanning from January 1, 2001, to December 31, 2021. In the bipolar disorder group, a total of 11,246 patients were recorded as being deceased throughout the study period. Moreover, the cause of death was determined using ICD-9-CM codes until December 31, 2014, and ICD-10-CM codes from January 1, 2015, onward from the National Cause of Death Registry. Suicide cases were identified using particular codes (ICD-9-CM codes E950–E959 and ICD-10-CM codes X60–X84 and Y87.0), with 1370 patients documented as having died by suicide.

### Definition of cases and controls

Individuals with bipolar disorder who died by suicide formed the case group. In this nested case–control study, these individuals were subdivided into five age groups: <30, 30–39, 40–49, 50–59, and ≥60 years. The date of the suicide death was designated as the index date. Using risk set sampling, controls were selected from living patients with bipolar disorder. In addition, 1 case was matched with up to 10 controls with the same sex and within a year of age (±1) on the index date and within the same year of initial diagnosis of bipolar disorder. The index date of their respective case was defined as the index date for controls. Cases identified later in the follow-up period were considered to be controls for preceding cases. This study’s sample comprised 1,370 cases and 13,700 controls.

### Covariates

This study thoroughly reviewed the NHIRD claims data of all cases and controls, including their demographic characteristics, health-care utilization patterns, psychiatric comorbidities, physical comorbidities, and prescribed medications. The demographic and clinical variables encompassed sex, age at baseline, index date, Charlson comorbidity index (CCI) score, urbanization level of the hospital’s location, and employment status. CCI was employed to assess the severity of physical comorbidities, and the scores of 0, 1, and ≥2 were assigned based on ICD-9-CM codes for primary and secondary diagnoses in the NHIRD [14, 15]. As outlined by Liu et al. [16], the urbanization of the regions of Taiwan was classified into five levels: highly urbanized: level 1, moderately urbanized: level 2, newly urbanized: level 3, township: level 4, and rural: level 5. Health-care utilization was evaluated by examining the proportion and frequency of subspecialty visits. Psychiatric and physical comorbidities were identified using ICD-9-CM and ICD-10 codes (e-Tables 1 and 2 in the Supplementary Material).

### Statistical analysis

The suicide mortality rate was computed by dividing the number of total new cases by the person-years contributed by each participant in the cohort. The cohort was classified into distinct age groups. Following a previously described methodology [2], the standardized mortality ratio (SMR) in this study was computed by comparing the observed mortality within the bipolar disorder group to the expected deaths in the general population of Taiwan from February 1, 2001, to December 31, 2021. The number of expected suicide mortality cases in the bipolar disorder group was calculated using sex-specific average mortality rates from the corresponding years in the general Taiwanese population. These rates were then multiplied by the person-years contributed by individuals diagnosed with bipolar disorder during the at-risk period.

In the nested case–control study design, univariable conditional logistic regression analysis was performed for each age group. In this analysis, factors such as demographic variables, health-care utilization, psychiatric and physical comorbidities in the 3 months leading up to suicide mortality were compared between cases and controls. Subsequently, the backward variable selection method was employed for multivariable analysis, and the models included psychiatric and physical comorbidities. Only variables exhibiting a significant association were retained in the final multivariable model (*P* < 0.01). Statistical analyses were conducted using SAS software (version 9.4; SAS Institute, Cary, NC, USA), with significance set at *P* < 0.01.

## Results

### Incidence and SMR stratified by age

**Figure 1.**
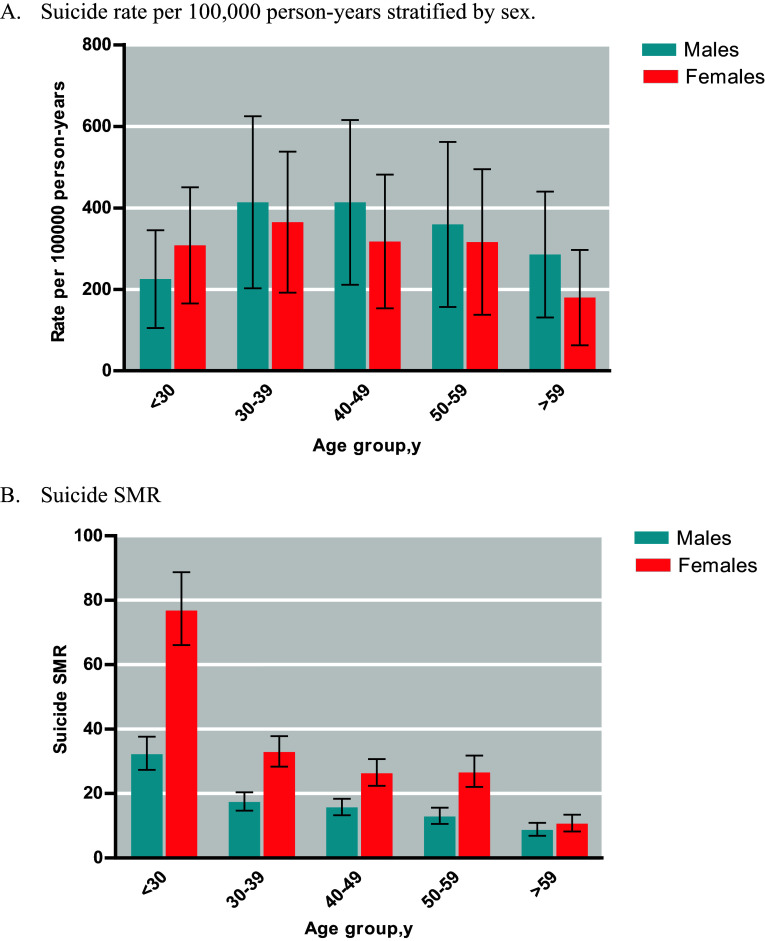
Incidence and standardized mortality ratio (SMR) of patients with bipolar disorder by age and sex. Error bars show 95% CI of incidence and SMR.

In the study cohort of 45,211 patients diagnosed with bipolar disorder, 1,370 individuals with bipolar disorder died by suicide during the follow-up period (e-Figure 1 in the Supplementary Material). The crude suicide rates (e-Table 3 in the Supplementary Material) were 165.2, 264.9, 253.0, 209.6, 151.0, and 85.2 per 100,000 person-years for individuals with bipolar disorder who were aged <30, 30–39, 40–49, 50–59, and ≥60 years, respectively. The SMR for suicide in the bipolar disorder group was 20.9 (95% CI, 19.8–14.9; *P* < 0.001). This finding indicates a significant disparity in suicide rates between individuals with bipolar disorder and the general population (Figure 1).

Age-stratified analyses revealed that all age groups had SMRs >1.00; SMRs progressively decreased with age. The highest SMR was observed in individuals with bipolar disorder aged <30 years (47.0; 95% CI, 42.1–52.2; *P* < 0.001), followed by those aged 30–39 years (23.8; 95% CI, 21.3–26.4; *P* < 0.001). Lower SMRs were found in older age groups. Additionally, in analyses stratified by age and sex, female patients aged <30 years had the highest SMR (76.8; 95% CI, 66.1–88.7; e-Table 3 in the Supplementary Material).

### Characteristics of cases and controls

Table 1 outlines the characteristics of patients who died by suicide and their controls. Most individuals who died by suicide were women (53.1%), and the proportion of suicide cases in each age group (i.e., <30, 30–39, 40–49, 50–59, and ≥ 60 years) ranged from 15.0 to 23.4%. Regarding urbanization levels, we observed that high proportions of both cases and controls resided in areas with level 1 urbanization (highly urbanized areas; 45.2% versus 47.6%) and level 2 urbanization (moderately urbanized areas; 28.6% versus 26.0%). Cases were more likely to be unemployed on the index date than controls (risk ratio = 1.50, *P* < 0.001).

**Table 1.** Demographic characteristics of cases and controls in a nested case–control study of a bipolar disorder cohort (ratio = 1:10; 1,370 cases and 13,700 controls)

	Cases (*N* = 1,370)	Controls (*N* = 13,700)	Crude	Model	
Sex	*N* (%)	*N* (%)	Risk ratio	95% CI	*P*-value
Male	643 (46.9)	6,430 (46.9)	Reference		
Female	727 (53.1)	7,270 (53.1)	–	–	–
Age at suicide mortality of cases and the corresponding index date of controls, years			–	–	–
<30	206 (15.0)	2,028 (14.8)	–	–	–
30–39	302 (22.0)	2,945 (21.5)	–	–	–
40–49	320 (23.4)	3,318 (24.2)	–	–	–
50–59	290 (21.2)	2,935 (21.4)	–	–	–
≥60	252 (18.4)	2,474 (18.1)	–	–	–
Charlson comorbidity index^a^					
0	821 (59.9)	8,189 (59.8)	Reference	–	–
1	311 (22.7)	2,840 (20.7)	1.09	0.95–1.25	0.247
≥2	238 (17.4)	2,671 (19.5)	0.87	0.74–1.03	0.105
Employment					
Yes	545 (39.8)	6,744 (49.2)	Reference		
No	825 (60.2)	6,956 (50.8)	1.50	1.33–1.68	<.001
Urbanization^a^					
Level 1	624 (45.6)	6,383 (46.6)	Reference		
Level 2	400 (29.2)	3,727 (27.2)	1.10	0.96–1.25	0.169
Level 3	57 (4.2)	840 (6.1)	0.69	0.52–0.92	0.010
Level 4	121 (8.8)	1,084 (7.9)	1.14	0.93–1.40	0.223
Level 5	168 (12.3)	1,666 (12.2)	1.03	0.86–1.24	0.738

a Level 1: highly urbanized, level 2: moderately urbanized, level 3, newly urbanized, level 4: township, level 5: rural.

The univariable regression results (Table 2) revealed that suicide was associated with several psychiatric disorders, including alcohol-induced mental disorders, drug-induced mental disorders, depressive episodes, anxiety states, personality disorders, and sleep disorders. Regarding physical comorbidities, other forms of heart disease, pneumonia, ulcer disease, and irritable bowel syndrome were associated with a high risk of suicide mortality.

**Table 2.** Psychiatric and physical comorbidities of cases and controls in a nested case–control study of a bipolar disorder cohort (ratio = 1:10; 3,848 cases and 13,700 controls)

	Cases	Controls			
	(*N* = 1,370)	(*N* = 13,700)	Crude	Model	
Within 3 months before the index date	*N* (%)	*N* (%)	Risk ratio	95% CI	*P*-value
Psychiatric comorbidities					
Dementia	78 (5.7)	660 (4.8)	1.21	0.94–1.56	0.135
Alcohol-induced mental disorders	97 (7.1)	522 (3.8)	2.01	1.59–2.54	<0.001
Drug-induced mental disorders	97 (7.1)	381 (2.8)	2.73	2.16–3.45	<0.001
Depressive episode	647 (47.2)	3,443 (25.1)	2.83	2.51–3.18	<0.001
Anxiety states	321 (23.4)	2,006 (14.6)	1.81	1.58–2.07	<0.001
Personality disorder	163 (11.9)	731 (5.3)	2.51	2.08–3.03	<0.001
Hyperactivity disorder	12 (0.9)	61 (0.5)	2.03	1.07–3.82	0.029
Intellectual disabilities	5 (0.4)	116 (0.9)	0.43	0.17–1.05	0.063
Sleep disorder	471 (34.4)	2,908 (21.2)	1.97	1.75–2.22	<0.001
Physical comorbidities					
Cardiovascular diseases					
Hypertension	217 (15.8)	2,230 (16.3)	0.96	0.82–1.14	0.651
Ischemic heart disease	55 (4.0)	595 (4.3)	0.92	0.69–1.22	0.560
Other forms of heart disease	110 (8.0)	551 (4.0)	2.13	1.71–2.64	<0.001
Congestive heart failure	25 (1.8)	200 (1.5)	1.26	0.83–1.93	0.285
Cerebrovascular diseases	64 (4.7)	591 (4.3)	1.09	0.83–1.44	0.523
Respiratory diseases					
Pneumonia	69 (5.0)	329 (2.4)	2.17	1.66–2.84	<0.001
COPD	55 (4.0)	502 (3.7)	1.11	0.83–1.48	0.499
Chronic bronchitis	40 (2.9)	310 (2.3)	1.32	0.93–1.85	0.116
Asthma	45 (3.3)	456 (3.3)	0.99	0.72–1.35	0.931
Upper respiratory tract infection	76 (5.6)	907 (6.6)	0.83	0.65–1.05	0.124
Gastrointestinal diseases					
Chronic hepatic disease	94 (6.9)	845 (6.2)	1.13	0.90–1.41	0.305
Ulcer disease	143 (10.4)	999 (7.3)	1.50	1.24–1.80	<0.001
Endocrine diseases					
Diabetes mellitus	145 (10.6)	1,567 (11.4)	0.91	0.76–1.10	0.324
Other diseases					
Cancer	58 (4.2)	474 (3.5)	1.24	0.94–1.64	0.136
Connective tissue disease	24 (1.8)	216 (1.6)	1.11	0.73–1.71	0.620
Moderate or severe renal disease	40 (2.9)	238 (1.7)	1.72	1.22–2.43	0.002
HIV infection	5 (0.4)	36 (0.3)	1.40	0.54–3.58	0.488
Atopic dermatitis and related conditions	11 (0.8)	94 (0.7)	1.17	0.63–2.19	0.621
Irritable bowel syndrome	40 (2.9)	231 (1.7)	1.76	1.25–2.48	0.001
Hyperlipidemia	116 (8.5)	1,314 (9.6)	0.87	0.71–1.06	0.164

### Demographic characteristics of cases and control groups and age subgroups

Table 3 presents the descriptive statistics of the demographic characteristics of the case and control groups and their five age subgroups. Notably, women were overrepresented in terms of suicide mortality in the age groups of <30, 40–49, and 50–59 years. CCI scores were not significantly associated with suicide in each age group.

**Table 3.** Demographics of cases and controls in a nested case–control study stratified by age groups

Age group, years	<30	<30	Risk ratio	30–39	30–39	Risk ratio	40–49	40–49	Risk ratio
	Cases	Control		Cases	Control		Cases	Control	
	(*N* = 206)	(*N* = 2,060)		(*N* = 302)	(*N* = 3,020)		(*N* = 320)	(*N* = 3,200)	
Sex	*n* (%)	*n* (%)		*n* (%)	*n* (%)		*n* (%)	*n* (%)	
Male	98 (47.6)	980 (47.6)	–	168 (55.6)	1,680 (55.6)	–	152 (47.5)	1,520 (47.5)	–
Female	108 (52.4)	1,080 (52.4)	–	134 (44.4)	1,340 (44.4)	–	168 (52.5)	1,680 (52.5)	–
CCI^a^									
0	154 (74.8)	1,627 (79.0)	Reference	210 (69.5)	2,145 (71.0)	Reference	218 (68.1)	2,043 (63.8)	Reference
1	35 (17.0)	308 (15.0)	1.20	72 (23.8)	564 (18.7)	1.30	67 (20.9)	662 (20.7)	0.95
≥2	17 (8.3)	125 (6.1)	1.45	20 (6.6)	311 (10.3)	0.65	35 (10.9)	495 (15.5)	0.66
Employment									
Yes	54 (26.2)	870 (42.2)	Reference	114 (37.8)	1,626 (53.8)	Reference	164 (51.3)	1,840 (57.5)	Reference
No	152 (73.8)	1,190 (57.8)	2.17**	188 (62.3)	1,394 (46.2)	1.92**	156 (48.8)	1,360 (42.5)	1.29
Urbanization									
1	114 (55.3)	953 (46.3)	Reference	140 (46.4)	1,447 (47.9)	Reference	137 (42.8)	1,445 (45.2)	Reference
2	63 (30.6)	658 (31.9)	0.80	94 (31.1)	840 (27.8)	1.16	90 (28.1)	842 (26.3)	1.13
3	2 (1.0)	129 (6.3)	0.13*	8 (2.7)	166 (5.5)	0.50	13 (4.1)	190 (5.9)	0.72
4	11 (5.3)	166 (8.1)	0.55	30 (9.9)	216 (7.2)	1.43	32 (10.0)	245 (7.7)	1.37
5	16 (7.8)	154 (7.5)	0.86	30 (9.9)	351 (11.6)	0.88	48 (15.0)	478 (14.9)	1.06

Age group, years	50–59	50–59	Risk ratio	> = 60	> = 60	Risk ratio			
	Cases	Control		Cases	Control				
	(*N* = 290)	(*N* = 2,900)		(*N* = 252)	(*N* = 2,520)				
Sex									
Male	123 (42.4)	1,230 (42.4)	–	136 (54.0)	1,360 (54.0)	–			
Female	167 (57.6)	1,670 (57.6)	–	116 (46.0)	1,160 (46.0)	–			
CCI^a^									
0	158 (54.5)	1,471 (50.7)	Reference	81 (32.1)	903 (35.8)	Reference			
1	70 (24.1)	715 (24.7)	0.91	67 (26.6)	591 (23.5)	1.27			
≥2	62 (21.4)	714 (24.6)	0.80	104 (41.3)	1,026 (40.7)	1.14			
Employment									
Yes	141 (48.6)	1,574 (54.3)	Reference	72 (28.6)	834 (33.1)	Reference			
No	149 (51.4)	1,326 (45.7)	1.26	180 (71.4)	1,686 (66.9)	1.24			
Urbanization									
1	131 (45.2)	1,380 (47.6)	Reference	102 (40.5)	1,158 (46.0)	Reference			
2	83 (28.6)	755 (26.0)	1.16	70 (27. 8)	632 (26.0)	1.26			
3	17 (5.9)	171 (5.9)	1.05	17 (6.8)	184 (7.3)	1.05			
4	27 (9.3)	248 (8.6)	1.15	21 (8.3)	209 (8.3)	1.14			
5	1 (11.0)	346 (11.9)	0.97	42 (16.7)	337 (13.4)	1.42			

a Charlson comorbidity index.

*P < 0.01, **P < 0.001.

Moreover, higher unemployment rates were found among cases than among controls in the <40 years age group, but not in the >40 years age group.

### Health-care utilization of cases and controls stratified by age

Table 4 presents findings on health-care utilization patterns in the 3 months preceding suicide in cases and controls across the age groups. Across all age groups, the majority of suicide cases had at least one outpatient visit, which was significantly more than that in living controls. Significantly higher risk ratios were found for subspecialty visits to psychiatry and emergency departments in cases than in controls across all age groups.

**Table 4.** Medical utilization within 3 months before suicide mortality for cases and controls in a nested case–control study stratified by age

Age group, years	<30	<30	Risk ratio	30–39	30–39	Risk ratio	40–49	40–49	Risk ratio
	Cases	Control		Cases	Control		Cases	Control	
	(*N* = 206)	(*N* = 2,060)		(*N* = 302)	(*N* = 3,020)		(*N* = 320)	(*N* = 3,200)	
	*n* (%)	*n* (%)		*n* (%)	*n* (%)		*n* (%)	*n* (%)	
All	190 (92.2)	1,749 (84.9)	2.22*	291 (96.4)	2,635 (87.3)	4.00**	310 (96.9)	2,882 (90.1)	3.48**
Psychiatric service	166 (80.6)	1,058 (51.4)	4.48**	251 (83.1)	1,734 (57.4)	4.07**	271 (84.7)	2,014 (62.9)	3.34**
Emergency visits	91 (44.2)	325 (15.8)	4.50**	140 (46.4)	480 (15.9)	4.79**	115 (35.9)	448 (14.0)	3.45**
Other service use									
Internal medicine	60 (29.1)	488 (23.7)	1.33	111 (36.8)	903 (29.9)	1.37	129 (40.3)	1,256 (39.3)	1.05
Surgery	29 (14.1)	166 (8.1)	1.85*	49 (16.2)	289 (9.6)	1.83**	43 (13.4)	403 (12.6)	1.08
Family medicine	47 (22.8)	392 (19.0)	1.27	78 (25.8)	728 (24.1)	1.10*	100 (31.3)	873 (27.3)	1.22
Pediatrics	14 (6.8)	168 (8.2)	0.82	17 (5.6)	226 (7.5)	0.74	17 (5.3)	197 (6.2)	0.85
Orthopedics	20 (9.7)	153 (7.4)	1.34	45 (14.9)	255 (8.5)	1.89**	39 (12.2)	368 (11.5)	1.07
Neurosurgery	6 (2.9)	31 (1.5)	1.97	9 (3.0)	68 (2.3)	1.33	15 (4.7)	98 (3.1)	1.55
Urology	8 (3.9)	36 (1.8)	2.25*	12 (4.0)	73 (2.4)	1.67	14 (4.4)	103 (3.2)	1.38
ENT	38 (18.5)	376 (18.3)	1.01	47 (15.6)	555 (18.4)	0.82	52 (16.3)	478 (14.9)	1.11
Ophthalmology	16 (7.8)	192 (9.3)	0.82	23 (7.6)	224 (7.4)	1.03	27 (8.4)	334 (10.4)	0.79
Dermatology	26 (12.6)	273 (13.3)	0.95	33 (10.9)	320 (10.6)	1.04	42 (13.1)	323 (10.1)	1.35
Neurology	15 (7.3)	64 (3.1)	2.40*	25 (8.3)	156 (5.2)	1.66*	25 (7.8)	226 (7.1)	1.12
Rehabilitation	11 (5.3)	79 (3.8)	1.42	14 (4.6)	116 (3.8)	1.21	23 (7.2)	155 (4.8)	1.52
Plastic surgery	12 (5.8)	21 (1.0)	6.21**	6 (2.0)	43 (1.4)	1.41	11 (3.4)	38 (1.2)	2.98*
Dentistry	42 (20.4)	457 (22.2)	0.90	58 (19.2)	574 (19.0)	1.01	51 (15.9)	634 (19.8)	0.77
Chinese herb medicine	40 (19.4)	386 (18.7)	1.05	62 (20.5)	555 (18.4)	1.15	57 (17.8)	583 (18.2)	0.97
Others	36 (17.5)	319 (15.5)	1.19	66 (21.9)	519 (17.2)	1.42*	54 (16.9)	552 (17.3)	0.97

Age group, years	50–59	50–59	Risk ratio	> = 60	> = 60	Risk ratio			
	Cases	Control		Cases	Control				
	(*N* = 290)	(*N* = 2,900)		(*N* = 252)	(*N* = 2,520)				
All	279 (96.2)	2,691 (92.8)	1.99	249 (98.8)	2,395 (95.0)	4.28			
Psychiatric service	239 (82.4)	1,900 (65.5)	2.46**	194 (77.0)	1,499 (59.5)	2.30**			
Emergency visits	104 (35.9)	383 (13.2)	3.66**	101 (40.1)	351 (13.9)	4.32**			
Other service use									
Internal medicine	130 (44.8)	1,425 (49.1)	0.84	166 (65.9)	1,507 (59.8)	1.30			
Surgery	47 (16.2)	389 (13.4)	1.25	39 (15.5)	363 (14.4)	1.09			
Family medicine	84 (29.0)	884 (30.5)	0.93	110 (43.7)	914 (36.3)	1.36			
Pediatrics	15 (5.17)	112 (3.9)	1.36	10 (4.0)	100 (3.97)	1.00			
Orthopedics	46 (15.9)	360 (12.4)	1.33	48 (19.1)	411 (16.3)	1.21			
Neurosurgery	14 (4.8)	118 (4.1)	1.20	17 (6.8)	114 (4.5)	1.52			
Urology	21 (7.2)	140 (4.8)	1.55	36 (14.3)	278 (11.0)	1.35			
ENT	41 (14.1)	471 (16.2)	0.85	46 (18.3)	361 (14.3)	1.33			
Ophthalmology	36 (12.4)	385 (13.3)	0.93	71 (28.2)	545 (21.6)	1.43			
Dermatology	17 (5.9)	292 (10.1)	0.56	30 (11.9)	287 (11.7)	1.05			
Neurology	28 (9.7)	312 (10.8)	0.89	52 (20.6)	464 (18.4)	1.16			
Rehabilitation	38 (4.8)	137 (4.3)	1.11	25 (9.9)	246 (9.8)	1.02			
Plastic surgery	9 (1.1)	20 (0.6)	1.80	6 (2.4)	22 (0.9)	2.76*			
Dentistry	56 (19.3)	627 (21.6)	0.87	48 (19.1)	504 (20.0)	0.94			
Chinese herb medicine	46 (15.9)	481 (16.6)	0.95	46 (18.3)	363 (14.4)	1.33			
Others	48 (16.6)	477 (16.5)	1.01	41 (16.3)	336 (13.3)	1.30			

*P < 0.01, **P < 0.001.

### Multivariable analysis

**Figure 2.**
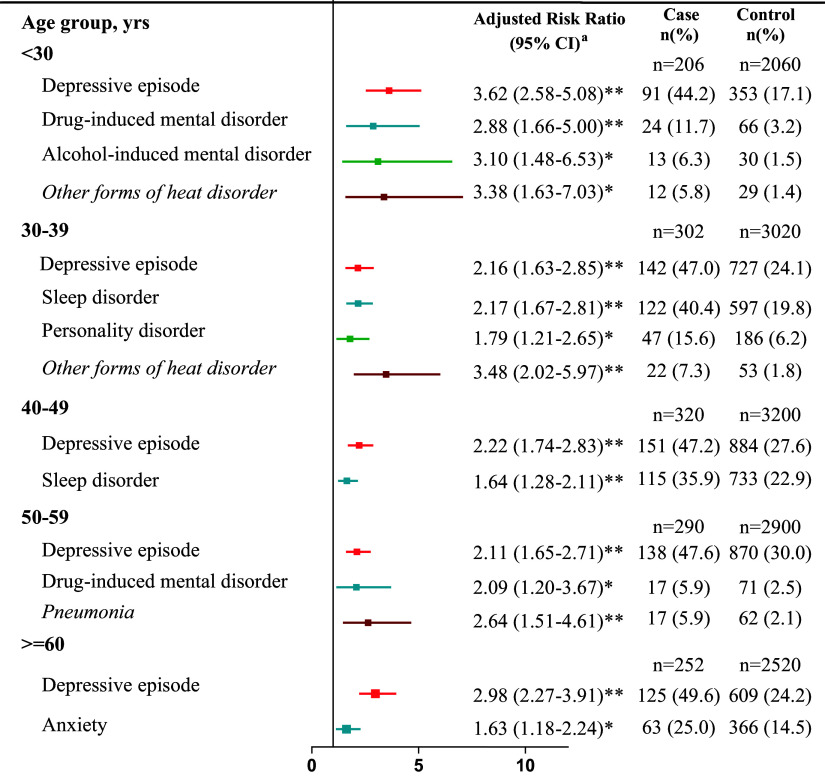
Multivariable conditional logistic regression of adjusted risk ratios of psychiatric and physical comorbidities in a nested case–control study stratified by age. Variables from e-Table 4 in the Supplementary Material remained in the multivariable conditional logistic regression model with *P* < 0.01 after stepwise selection. *P < 0.01; **P < 0.001.

The distribution of psychiatric and physical comorbidities and the results of univariable regression analyses are presented in e-Table 4 in the Supplementary Material. The results of the multivariable analysis are presented in Figure 2 and e-Table 5 in the Supplementary Material.

Regarding psychiatric comorbidities, the prevalence of depressive episodes was significantly higher among cases than among controls across all age groups. Moreover, increased risks of drug-induced and alcohol-induced mental disorders were prevalent in the <30 years age group for suicide cases, and drug-induced mental disorder was prevalent in the 50–59 years age group. Sleep disorders were associated with suicide mortality in the 30–49 years age group, and personality disorder was associated with suicide in the 30–39 years age group. Anxiety disorder was associated with suicide in the ≥60 years age group.

Regarding physical comorbidities, other forms of heart disease were associated with suicide in the age group of <40 years. Pneumonia was associated with a high risk of suicide in the 50–59 years age group.

## Discussion

### Major findings

To the best of our knowledge, this population-based cohort study is the first to examine age-specific risk profiles for suicide mortality throughout the lifespan of patients with bipolar disorder. The SMRs ranged from 9.5 to 47.0 across the age groups. Moreover, young female patients with bipolar disorder who were aged <30 years had the highest SMR (76.8).

### Incidence and SMR

This study found that young patients with bipolar disorder who were aged <30 years had the highest SMR, which accords with the findings of previous studies [3, 5]. Young patients with early-onset bipolar disorder often experience a prolonged and severe disease course, with persistent symptoms and infrequent remission. Compared with adults, young patients with bipolar disorder experience significantly more mixed and cycling episodes and frequent mood polarity shifts per year [17]. Additionally, they are more vulnerable to the compounded effects of stressors, including difficulties in interpersonal relationships, academic challenges, and unfavorable job conditions, which may increase their risk of suicide mortality [17].

Globally, men have higher suicide rates than women, with approximately three of four deaths by suicide occurring among men [4]. In the general population of Taiwan, a significantly higher incidence of suicide mortality was found in male patients than in female patients [1]. The present study observed that young female patients aged <30 years had the highest SMR. Moreover, this study revealed that the incidence of suicide mortality was higher in female patients aged <30 years than in their male counterparts. This finding suggests that young female patients with bipolar disorder have an increased risk of suicide; hence, careful attention should be paid to these patients. Further research should be conducted to reveal the potential reasons for these findings. Women with bipolar disorder may be more vulnerable to psychosocial stressors than men with bipolar disorder [2]. Although our study focused on young female patients (<30 years) because of the exceptionally high SMR (76.8) found in this age group, the data reveal that women in other age groups also exhibit increased suicide mortality rates compared with men. This broader age group of female patients affected by suicide risk suggests that gender-related vulnerabilities in bipolar disorder extend beyond early adulthood. Studies have suggested that biologically, women with bipolar disorder are more susceptible to inflammatory and metabolic disruptions than their male counterparts [17]. These disruptions may in turn increase their risks of psychiatric and physical comorbidities [18], potentially increasing their suicide risk.

### Employment status

In this study, the employment rate in the control group (patients with bipolar disorder without suicide mortality) ranged from 33.1% (aged >60 years) to 54.3% (50–59 years). This finding is consistent with that of previous studies, which have found employment rates of 40–60% in patients with bipolar disorder [19] and only 10–30% in patients with schizophrenia [20].

A major finding of the present study is the significant association between unemployment and suicide risk only in patients younger than 40 years. Holm et al. [21] observed an employment rate of approximately 45% in the 3 years before the diagnosis of bipolar disorder and a decline in this employment rate after 1 year from the diagnosis. Thus, monitoring the employment status of patients is crucial, and vocational rehabilitation must be provided during the first few years after bipolar disorder onset.

Young patients with bipolar disorder who are unemployed may struggle with financial difficulties and low self-esteem, making them vulnerable to depression; depression development can increase the risk of suicide among these patients [2, 22]. Patients with bipolar disorder aged >40 years, although unemployed, may have become accustomed to their condition or are receiving social welfare assistance, which may lead to a decreased risk of suicide.

### Health-care utilization

A previous study reported that individuals who died by suicide had higher rates of health-care utilization across all ages. Moreover, before suicide, a high relative risk and a high absolute health-care utilization rate (43.8%) for emergency room services were found [23]. In this study, we reported that in each age group, patients with bipolar disorder tended to exhibit increased visits to the emergency department and increased utilization of psychiatric services separately within 3 months before suicide mortality. This study revealed that most cases and controls resided in areas with high or moderate urbanization levels. Urban stressors, such as high living costs, crowded living conditions, and fast-paced lifestyles, may negatively affect mental health and increase suicide risk in patients with bipolar disorder, potentially leading to their increased health-care utilization [24, 25]. Our findings suggest that patients living in urban areas have greater access to psychiatric services and emergency departments than do their counterparts residing in rural areas; this is possibly due to the greater availability of specialized care in urban areas, which results in differences in health-seeking behaviors between urban and rural populations. These observations underscore the importance of reducing barriers to health care in order to improve healthcare accessibility.

Patients with bipolar disorder who died by suicide may have had unstable mental states (such as depressive episodes) before their death; thus, they are more likely to seek psychiatric care or to visit the emergency department. A previous suicide attempt is widely recognized as one of the most significant predictors of suicide death across all populations [4]. Studies [6, 26] have also revealed that a previous suicide attempt is a significant predictor of death by suicide in patients with bipolar disorder. Those who have attempted suicide may require visits to the emergency department for psychiatric crisis intervention and injury care.

In summary, increased use of psychiatric and emergency services before suicide may reflect underlying psychiatric and physical comorbidities, so targeted interventions are needed for prevention.

### Psychiatric comorbidities

Intriguingly, one of the major findings in this study showed that psychiatric comorbidity contributes to suicide mortality more than physical comorbidity. Several psychiatric comorbidities were identified to be significantly associated with suicide in various age subgroups.

This study observed that in each age group, a depressive episode of bipolar disorder was associated with suicide. Additionally, suicide attempts lead to more lethal consequences in individuals with bipolar disorder than in those with other psychiatric conditions [27]. The attempt-to-completion ratio is 3:1 in patients with bipolar disorder compared with 35:1 in the general population [28]. This observation highlights the high burden of depression in patients with bipolar disorder and the increased lethality of suicide attempts, which contribute to increased suicide rates [9]. Considering this association, early identification of and targeted intervention for depressive episodes are critical for reducing the risk of suicide in patients with bipolar disorder.

This study determined that drug- and alcohol-induced mental disorders were linked to suicide mortality in individuals with bipolar disorder aged <30 years. Additionally, drug-induced disorders were a risk factor for those aged 50–59 years. Therefore, drug-induced mental disorders were associated with increased suicide risk in both individuals aged <30 years and those aged 50–59 years. In the younger group, drug-induced mental disorders were linked to the use of illicit drugs, and in the older group, these disorders may be associated with prescribed medications, such as benzodiazepines.

A previous study revealed a higher prevalence of substance use, particularly the use of highly addictive and hallucinogenic substances such as cocaine, amphetamines, opioids, cannabis, and hallucinogens, in individuals with bipolar disorder than in those with schizophrenia [29]. Ernst et al. [30] observed that 46% of patients had bipolar disorder onset before the age of 19 years and that early onset before the age of 19 years was associated with comorbid substance abuse. Young patients with bipolar disorder tend to exhibit impulsive behaviors, and this impulsivity is closely associated with the prevalence of substance use disorders, both of which are indicators of suicide risk [31–33].

Few studies have explored whether sleep disorders are a risk factor for suicide across different age groups of patients with bipolar disorder. A study revealed a correlation between nightmares and suicide in patients with bipolar disorder in the 6–15 years age group [34]. The present study demonstrated that sleep disorders were associated with suicide risk in the 30–49 years age group, but not in the <30 age group. This may be because individuals with bipolar disorder aged 30 to 49 years are in their working years, and sleep disorders during this period exert additive effects with stress responses, thereby increasing their risk of suicide. However, further research is required to verify this hypothesis.

A study demonstrated that anxiety disorders increase the risk of suicide [35]. Individuals with bipolar disorder are more likely to have anxiety disorders than the general population, and approximately 45% of these patients exhibit comorbid anxiety disorders [36]. In this study, we identified that anxiety was associated with suicide only in older patients with bipolar disorder. We deduced that anxiety disorders are the most common mental disorder in later life [37]. Moreover, older patients with bipolar disorder have a higher likelihood of suicide than younger individuals [38].

Univariable analysis (e-Table 4 in the Supplementary Material) revealed a significant association between anxiety and suicide in individuals aged <30 years. However, in multivariable analysis, the association between anxiety and suicide did not remain significant in the model, as factors such as depressive episodes, drug-induced disorders, and alcohol-induced disorders outweighed the suicide risk.

### Physical comorbidities

Patients with bipolar disorder have a higher rate of comorbidities than the general population or individuals with schizophrenia [39, 40]. However, in this study, we found that physical comorbidities had lower effects on suicide, and only other forms of heart disease in the <40 years age group and pneumonia in the 50–59 years age group were associated with an increased risk of suicide.

Bipolar disorder is strongly associated with an increased risk of cardiovascular diseases (e.g., other forms of heart disease and ischemic heart disease) [39], which comprise a major cause of death in patients with bipolar disorder [12]. Patients with cardiovascular diseases have a lower quality of life or have comorbid depression, increasing their risk of suicide.

A large-scale Denmark study [41] revealed that patients who had been hospitalized with an infection had a 40% increased risk of suicide. For patients with seven or more infections, the risk of suicide increased by nearly 300%, indicating a dose–response relationship. This finding reinforces the causal relationship of infections with suicide. These findings suggest that inflammation may play a relevant role in the pathological mechanisms underlying suicidal behavior. However, in this study, pneumonia was associated with an increased risk of suicide only in the 50–59 years age group. The underlying reason remains to be investigated.

### Strengths and limitations

This study has several strengths, including the use of a nationwide cohort of bipolar disorder patients and a sufficient number of suicide cases. Furthermore, we employed a nested case–control study design, which reflects the circumstances leading to suicide, facilitating the investigation of age-stratified short-term risk profiles before the event.

However, this study has several limitations. First, this study did not examine certain variables that were potentially associated with suicide, such as lifestyle factors or previous suicide attempts. Second, competing risks may occur. Some patients with bipolar disorder could have died from causes other than suicide, which would have excluded them from being at risk of subsequent suicide mortality during the study period. Third, we included only patients with bipolar disorder who had been hospitalized, which limits the generalizability of the findings to the entire population of patients with bipolar disorder.

Finally, this study offers valuable insights into suicide risk in patients with bipolar disorder. However, further research is required on this topic. Given the limitations of the age-stratified nested case–control study design, this study examined comorbidities within the 3 months preceding suicide mortality rather than identifying temporal trends. Future larger longitudinal studies should be conducted to explore the progression of mental health conditions and their association with suicide risk. Additional studies should explore how substance use contributes to suicide mortality; these insights may aid in the development of targeted strategies for suicide prevention in patients with bipolar disorder.

### Implications

This study found increased suicide risks across all age groups of patients with bipolar disorder, especially among younger individuals. Vocational rehabilitation is crucial for suicide prevention among those aged <40 years. Various risk factors for suicide were identified across different age groups. Based on these insights, enhanced health-care intervention strategies should be developed for preventing suicide in patients with bipolar disorder in different life stages.

## Supporting information

Lin et al. supplementary material Lin et al. supplementary material

## Data Availability

Data sharing is not applicable to this article as the national registry raw data is not accessible due to privacy protection regulations.

## Supplementary material

The supplementary material for this article can be found at http://doi.org/10.1192/j.eurpsy.2025.2451.

## References

[1] Pan YJ, Yeh LL, Chan HY, Chang CK. Excess mortality and shortened life expectancy in people with major mental illnesses in Taiwan. Epidemiol Psychiatr Sci. 2020;29:e156. 10.1017/s2045796020000694. 32792024 PMC7443795

[2] Chen PH, Tsai SY, Pan CH, Chen YL, Chang HM, Su SS, et al. Sex-specific risk profiles for suicide mortality in bipolar disorder: incidence, healthcare utilization and comorbidity. Psychol Med. 2023;53(4):1500–9. 10.1017/s003329172100307x. 34779754

[3] Pan YJ, Yeh LL, Chan HY, Chang CK. Transformation of excess mortality in people with schizophrenia and bipolar disorder in Taiwan. Psychol Med. 2017;47(14):2483–93. 10.1017/S0033291717001040. 28443526

[4] World Health Organization. Fact sheet of suicide. 2023. https://www.who.int/news-room/fact-sheets/detail/suicide.

[5] Osby U, Brandt L, Correia N, Ekbom A, Sparén P. Excess mortality in bipolar and unipolar disorder in Sweden. Arch Gen Psychiatry. 2001;58(9):844–50. 10.1001/archpsyc.58.9.844. 11545667

[6] Tsai SY, Kuo CJ, Chen CC, Lee HC. Risk factors for completed suicide in bipolar disorder. J Clin Psychiatry. 2002;63(6):469–76. 10.4088/jcp.v63n0602. 12088157

[7] Hoye A, Nesvag R, Reichborn-Kjennerud T, Jacobsen BK. Sex differences in mortality among patients admitted with affective disorders in North Norway: a 33-year prospective register study. Bipolar Disord. 2016;18(3):272–81. 10.1111/bdi.12389. 27226265

[8] Høyer EH, Mortensen PB, Olesen AV. Mortality and causes of death in a total national sample of patients with affective disorders admitted for the first time between 1973 and 1993. Br J Psychiatry. 2000;176:76–82. 10.1192/bjp.176.1.76. 10789332

[9] Miller JN, Black DW. Bipolar disorder and suicide: a review. Curr Psychiatry Rep. 2020;22(2):6. 10.1007/s11920-020-1130-0. 31955273

[10] Conwell Y, Duberstein PR, Cox C, Herrmann JH, Forbes NT, Caine ED. Relationships of age and axis I diagnoses in victims of completed suicide: a psychological autopsy study. Am J Psychiatry. 1996;153(8):1001–8. 10.1176/ajp.153.8.1001. 8678167

[11] Cheng AT. Mental illness and suicide. A case-control study in east Taiwan. Arch Gen Psychiatry. 1995;52(7):594–603. 10.1001/archpsyc.1995.03950190076011. 7598636

[12] Chen PH, Tsai SY, Chen PY, Pan CH, Su SS, Chen CC, et al. Lipid-modifying agents and risk of all-cause, natural and suicide mortality in schizophrenia: nationwide cohort study. Br J Psychiatry. 2024:1–9. 10.1192/bjp.2024.85. 38751180

[13] Tsai SJ, Cheng CM, Chang WH, Bai YM, Hsu JW, Huang KL, et al. Risks and familial coaggregation of death by suicide, accidental death and major psychiatric disorders in first-degree relatives of individuals who died by suicide. Br J Psychiatry. 2023;223(4):465–70. 10.1192/bjp.2023.85. 37350338 PMC10866671

[14] Charlson ME, Pompei P, Ales KL, MacKenzie CR. A new method of classifying prognostic comorbidity in longitudinal studies: development and validation. J Chronic Dis. 1987;40(5):373–83. 10.1016/0021-9681(87)90171-8. 3558716

[15] Deyo RA, Cherkin DC, Ciol MA. Adapting a clinical comorbidity index for use with ICD-9-CM administrative databases. J Clin Epidemiol. 1992;45(6):613–9. 10.1016/0895-4356(92)90133-8. 1607900

[16] Liu CY, Hung YT, Chuang YL, Chen YJ, Weng WS, Liu JS, Liang KY. Incorporating development stratification of Taiwan townships into sampling design of large scale health interview survey. J Health Manage. 2006;4(1):1–22.

[17] Baskaran A, Cha DS, Powell AM, Jalil D, McIntyre RS. Sex differences in rates of obesity in bipolar disorder: postulated mechanisms. Bipolar Disord. 2014;16(1):83–92. 10.1111/bdi.12141. 24467470

[18] Crump C, Sundquist K, Winkleby MA, Sundquist J. Comorbidities and mortality in bipolar disorder: a Swedish national cohort study. JAMA Psychiatry. 2013;70(9):931–9. 10.1001/jamapsychiatry.2013.1394. 23863861

[19] Marwaha S, Durrani A, Singh S. Employment outcomes in people with bipolar disorder: a systematic review. Acta Psychiatr Scand. 2013;128(3):179–93. 10.1111/acps.12087. 23379960

[20] Haro JM, Novick D, Bertsch J, Karagianis J, Dossenbach M, Jones PB. Cross-national clinical and functional remission rates: Worldwide Schizophrenia Outpatient Health Outcomes (W-SOHO) study. Br J Psychiatry. 2011;199(3):194–201. 10.1192/bjp.bp.110.082065. 21881098

[21] Holm M, Taipale H, Tanskanen A, Tiihonen J, Mitterdorfer-Rutz E. Employment among people with schizophrenia or bipolar disorder: a population-based study using nationwide registers. Acta Psychiatr Scand. 2021;143(1):61–71. 10.1111/acps.13254. 33155273 PMC7839734

[22] Rajewska-Rager A, Sibilski P, Lepczyńska N. Risk factors for suicide among children and youths with bipolar spectrum and early bipolar disorder. Psychiatr Polska. 2015;49(3):477–88. 10.12740/pp/29415. 26276916

[23] Ahmedani BK, Westphal J, Autio K, Elsiss F, Peterson EL, Beck A, et al. Variation in patterns of health care before suicide: a population case-control study. Prev Med. 2019;127:105796. 10.1016/j.ypmed.2019.105796. 31400374 PMC6744956

[24] Montanari A, Wang L, Birenboim A, Chaix B. Urban environment influences on stress, autonomic reactivity and circadian rhythm: protocol for an ambulatory study of mental health and sleep. Front Public Health. 2024;12:1175109. 10.3389/fpubh.2024.1175109. 38375340 PMC10875008

[25] Cyr ME, Etchin AG, Guthrie BJ, Benneyan JC. Access to specialty healthcare in urban versus rural US populations: a systematic literature review. BMC Health Serv Res. 2019;19(1):974. 10.1186/s12913-019-4815-5. 31852493 PMC6921587

[26] Simon GE, Hunkeler E, Fireman B, Lee JY, Savarino J. Risk of suicide attempt and suicide death in patients treated for bipolar disorder. Bipolar Disord. 2007;9(5):526–30. 10.1111/j.1399-5618.2007.00408.x. 17680924

[27] Dennehy EB, Marangell LB, Allen MH, Chessick C, Wisniewski SR, Thase ME. Suicide and suicide attempts in the Systematic Treatment Enhancement Program for Bipolar Disorder (STEP-BD). J Affect Disord. 2011;133(3):423–7. 10.1016/j.jad.2011.04.036. 21601286 PMC3163014

[28] Plans L, Barrot C, Nieto E, Rios J, Schulze TG, Papiol S, et al. Association between completed suicide and bipolar disorder: a systematic review of the literature. J Affect Disord. 2019;242:111–22. 10.1016/j.jad.2018.08.054. 30173059

[29] Maremmani AG, Bacciardi S, Gehring ND, Cambioli L, Schütz C, Jang K, et al. Substance use among homeless individuals with schizophrenia and bipolar disorder. J Nerv Mental Dis. 2017;205(3):173–7. 10.1097/nmd.0000000000000462. 26785056

[30] Ernst CL, Goldberg JF. Clinical features related to age at onset in bipolar disorder. J Affect Disord. 2004;82(1):21–7. 10.1016/j.jad.2003.10.002. 15465573

[31] Grant BF, Stinson FS, Dawson DA, Chou SP, Dufour MC, Compton W, et al. Prevalence and co-occurrence of substance use disorders and independent mood and anxiety disorders: results from the National Epidemiologic Survey on Alcohol and Related Conditions. Arch Gen Psychiatry. 2004;61(8):807–16. 10.1001/archpsyc.61.8.807. 15289279

[32] Dalton EJ, Cate-Carter TD, Mundo E, Parikh SV, Kennedy JL. Suicide risk in bipolar patients: the role of co-morbid substance use disorders. Bipolar Disord. 2003;5(1):58–61. 10.1034/j.1399-5618.2003.00017.x. 12656940

[33] Swann AC, Dougherty DM, Pazzaglia PJ, Pham M, Moeller FG. Impulsivity: a link between bipolar disorder and substance abuse. Bipolar Disord. 2004;6(3):204–12. 10.1111/j.1399-5618.2004.00110.x. 15117399

[34] Stanley IH, Hom MA, Luby JL, Joshi PT, Wagner KD, Emslie GJ, et al. Comorbid sleep disorders and suicide risk among children and adolescents with bipolar disorder. J Psychiatr Res. 2017;95:54–9. 10.1016/j.jpsychires.2017.07.027. 28777984 PMC5653415

[35] Khan A, Leventhal RM, Khan S, Brown WA. Suicide risk in patients with anxiety disorders: a meta-analysis of the FDA database. J Affect Disord. 2002;68(2–3):183–90. 10.1016/s0165-0327(01)00354-8. 12063146

[36] Pavlova B, Perlis RH, Alda M, Uher R. Lifetime prevalence of anxiety disorders in people with bipolar disorder: a systematic review and meta-analysis. Lancet Psychiatry. 2015;2(8):710–7. 10.1016/s2215-0366(15)00112-1. 26249302

[37] Andreas S, Schulz H, Volkert J, Dehoust M, Sehner S, Suling A, et al. Prevalence of mental disorders in elderly people: the European MentDis_ICF65+ study. Br J Psychiatry. 2017;210(2):125–31. 10.1192/bjp.bp.115.180463. 27609811

[38] Voshaar RC, van der Veen DC, Kapur N, Hunt I, Williams A, Pachana NA. Suicide in patients suffering from late-life anxiety disorders; a comparison with younger patients. Int Psychogeriatr. 2015;27(7):1197–205. 10.1017/s1041610215000125. 25669916

[39] Chen PH, Tsai SY, Pan CH, Chen YL, Su SS, Chen CC, et al. Prevalence and 5-year trend of incidence for medical illnesses after the diagnosis of bipolar disorder: a nationwide cohort study. Aust N Z J Psychiatry. 2022;56(9):1164–76. 10.1177/00048674211046891. 34558298

[40] Chen YL, Chen PY, Pan CH, Chen PH, Su SS, Tsai SY, et al. Prevalence and 3-year incidence of physical illnesses after schizophrenia diagnosis: comparison with general population. Schizophr Res. 2024;264:272–9. 10.1016/j.schres.2024.01.009. 38198879

[41] Lund-Sorensen H, Benros ME, Madsen T, Sorensen HJ, Eaton WW, Postolache TT, et al. A nationwide cohort study of the association between hospitalization with infection and risk of death by suicide. JAMA Psychiatry. 2016;73(9):912–9. 27532502 10.1001/jamapsychiatry.2016.1594

