# Supplementary material for: Risk profiles before suicide mortality in patients with bipolar disorder across the lifespan
Source: Eur Psychiatry. 2025 Apr 21;68(1):e59. doi: 10.1192/j.eurpsy.2025.2451 (PMC12188330; doi:10.1192/j.eurpsy.2025.2451)
Supplement: Lin et al. supplementary material [file S0924933825024514sup001.docx]

**Supplementary Materials**

**List of e-Tables and figures**

**e-Figure 1.** Study flowchart

**e-Table 1.** ICD-9-CM codes for physical and psychiatric comorbidities

**e-Table 2.** ICD-10-CM codes for physical and psychiatric comorbidities

**e-Table 3.** Standardized mortality ratios of suicide mortality among patients with bipolar disorder stratified by sex and age at baseline (cohort, N = 45211; mortality, n = 11246; suicide mortality, n = 1370)

**e-Table 4.** Univariable conditional logistic regression for calculating the adjusted risk ratios of psychiatric and physical comorbidities in a nested case–control study stratified by age (variables with P < 0.01 were included in the model).

**e-Table 5**. Multivariable conditional logistic regression for calculating the adjusted risk ratios of psychiatric and physical comorbidities in a nested case–control study stratified by age (variables with P < 0.01 were included in the model)

**e-Figure 1.** Study flowchart

Patients with mental disorder (ICD-9-CM code of 290.**–319.**) between January 1, 2000, and December 31, 2021 (n = 12,829,791) are included

(1) Patients with at least one psychiatric admission with a discharge diagnosis of bipolar disorder (ICD-9-CM codes 296.0 to 296.16, 296.4 to 296.81, 296.89, 296.9; ICD-10 codes: F30.x, F31.x) between January 1, 2000, and December 31, 2021, are included.

(2) The earliest (or first) admission (above) is used as the index admission (the baseline).

(N = 45,628)

Patients ever diagnosed with schizophrenia (ICD-9-CM codes 295.**; ICD-10-CM codes F20.*, F25.*) in the study cohort between January 1, 2000, and December 31, 2021 (N = 315,088) are excluded

Patients with bipolar disorder in our cohort are identified and included

(*n* =45211).

Patients with mortality (*n* = 11246) and suicide (*n* = 1370) at any time between January 1, 2001, and December 31, 2021.

Patients with missing data (gender or age) are excluded (*n* = 417).

Patient data linked with national mortality database.

1370 valid case–control pairs are formed (case = 1370, control = 13,700)

10 controls for each case, matched by sex, age (±1), and the year of first bipolar disorder diagnosis, are drawn from the general population.

**e-Table 1.** ICD-9-CM codes for physical and psychiatric comorbidities

| **Physical comorbidities** | *ICD-9-CM* codes |
| --- | --- |
| Cardiovascular diseases |  |
| Hypertension | 401–405 |
| Ischemic heart disease | 410–414 |
| Congestive heart failure | 428, 429.3, 402.01, 402.11, 402.91, 404.01, 404.03, 404.11, 404.13, 404.91, 404.93 |
| Other forms of heart disease | 420–429 |
| Diseases of arteries, arterioles, and capillaries | 440–449 |
| Diseases of veins and lymphatics | 451–459 |
| Cerebrovascular diseases | 430–438 |
| Respiratory diseases |  |
| URI | 460 |
| Pneumonia | 480–486, 507 |
| Asthma | 493 |
| Chronic bronchitis | 491 |
| COPD | 490, 491, 492, 496, A323, A325 |
| Tuberculosis | 010–018 |
| Gastrointestinal diseases |  |
| Chronic hepatic disease | 571 |
| Ulcer | 531–534 |
| Irritable bowel syndrome | 564.1 |
| Renal diseases |  |
| Chronic kidney disease and renal failure | 585, 586, V56, V420, V451, 39.27, 39.42, 39.93, 39.94, 39.95, 54.98 |
| Endocrine/metabolic diseases |  |
| Diabetes mellitus | 250 |
| Hyperlipidemia | 272 |
| Musculoskeletal/integument diseases |  |
| Connective tissue disease | 710, 714 |
| Atopic dermatitis | 691 |
| HIV infection | 042, 043, 044 |
| Cancer | 140–209 |
| **Psychiatric comorbidities** | *ICD-9-CM* codes |
| Depressive episodes | 2962、2963、3004、311 |
| Substance use-related disorder | 292、304、305 (exclude 3050) |
| Alcohol use-related disorder | 291、303、3050 |
| Neurocognitive disorder | 294、290 |
| Sleep disorder | 3074、7805、327 |
| Anxiety disorder | 3000、3009 |
| Hyperactivity disorder | 314 |
| Personality disorder | 301 |
| Intellectual disabilities | 317、318、319 |

Abbreviations: COPD = chronic obstructive pulmonary disease, HIV = human immunodeficiency virus, URI = upper respiratory tract infection

**e-Table 2.** ICD-10-CM codes for physical and psychiatric comorbidities

| **Physical comorbidities** | *ICD-10-CM* codes |
| --- | --- |
| Cardiovascular Diseases |  |
| Hypertension | I10、I110、I119、I129、I120、I1310、I130、I1311、I132 |
| Congestive heart failure | I110、I130、I132、I420、I421、I422、I423、I424、I425、I426、I427、I428、I429、I501、I509、I517' I5020、I5021、I5022、I5023、I5030、I5031、I5032、I5033、I5040、I5041、I5042、I5043、I43 |
| Other forms of heart disease | I301、I309、I300、I308、I330、 I339、 I409、I400、I401、I408、I312、I310、I311、 I318、I313、I314、I319、I340、I341、I342、I348、I349、I350、I351、I352、I358、I359、I360、I361、I362、I368、I369、I370、I371、I372、I378、I379、 I423、I421、I428、I424、I420、I422、I425、I429、I426、I427、I442、I443、I440、I441、I444、I445、I446、I447、I450、I451、I454、I452、I453、I455、I456、I458、I459、I471、I492、I470、I472、I493、I479、I480、I482、I489、I481、I483、I484、I490、I462、I468、I469、I494、I491、I495、R001、I498、I499、I502、I503、I504、I509、I501、I514、I32、I41、I38、I43、I39 |
| Diseases of arteries, arterioles, and capillaries | I700、I701、I758、I702、I709、I750、I703、I706、I707、I704、I705、I708、I710、I711、I712、I713、I714、I718、I715、I716、I719、I778、I790、I721、I722、I777、I723、I724、I720、I728、I729、I730、I731、I791、I798、I738、I739、I740、I741、I742、I743、I744、I745、I748、I749、M300、M302、M308、M317、M303、M310、M312、M301、M313、M315、M316、M311、M314、I770、I771、I772、I773、I774、I775、M318、M319、I776、I779、I780、I781、I788、I789 |
| Diseases of veins and lymphatics | I800、I801、I802、I803、I808、I809、I81、I820、I821、I822、I823、I824、I825、I826、I827、I828、I829、I82A、I82B、I82C、I830、I831、I832、I838、I839、K640、K641、K642、K643、K648、K644、K645、K649、I850、I851、I860、I861、I862、I863、I864、I868、I972、I890、I891、I898、I899、I951、I950、I958、I952、I953、I959、R58 |
| Cerebrovascular diseases | I60、I61、I62、I65、I63、I66、G45、G46、I67 |
| Respiratory diseases |  |
| URI | J00 |
| Pneumonia | J14、J17、J13、J120、J121、J122、J123、J128、J128、J129、J181、J150、J151、J154、J154、J153、J154、J152、J152、J152、J158、J155、J156、A481、J158、J159、J157、J160、J168、B250、A379、A221、B440、J180、J188、J189、J690、J691、J698 |
| Asthma | J452、J453、J454、J455、J459、J449、J440、J441 |
| Chronic bronchitis | J42、J410、J411、J418、J449、J441、J440 |
| COPD | J410、J411、J449、J440、J441、J418、J439、J430、J431、J432、J438、J439、J449 |
| Gastrointestinal diseases |  |
| Chronic hepatic disease | K70、K73、K75、K74、K76、R16 |
| Ulcer disease | K25、K31、K56、K26、K27、K28 |
| Irritable bowel syndrome | K580、K589 |
| Renal diseases |  |
| Chronic kidney disease and renal failure | N184、N185、N186、N189 Procedures code (0313、0314、0315、0316、0317、0318、0319、031A、031B、031C、0312、03WY、03PY、5A1D、3E1M) |
| Endocrine/metabolic diseases |  |
| Hyperlipidemia | E780、E781、E782、E783、E784、E785、E786、E881、E752、E753、E770、E771、E778、E779、E713、E755、E787、E788、E882、E888、E756、E789 |
| Diabetes mellitus | E119、E139、E109、E116、E106、E101、E080、E090、E110、E130、E086、E096、E131、E136、E112、E132、E102、E083、E093、E113、E133、E103、E084、E094、E114、E134、E104、E085、E095、E115、E135、E105、E088、E098、E118、E138、E108 |
| Musculoskeletal/integument diseases |  |
| Connective tissue disease | M32、M34、M35、M33、M36、M05、M06、M08、M12 |
| Atopic dermatitis | L22、L200、L208、L209 |
| HIV infection | B20 |
| Cancer | C01、C07、C12、C19、C20、C23、C33、C37、C55、C58、C52、C61、C73、D45、C000、Z511、C001、C003、C004、C005、C006、C008、C002、C009、C020、C021、C022、C023、C028、C024、C029、C080、C081、C089、C030、C031、C039、C040、C041、C048、C049、C060、C061、C050、C051、C052、C058、C059、C062、C068、C069、C098、C099、C090、C091、C100、C101、C108、C102、C103、C104、C109、C110、C111、C112、C113、C118、C119、C130、C131、C132、C138、C139、C140、C142、C148、C153、C154、C155、C158、C159、C160、C7A0、C164、C163、C161、C162、C165、C166、C168、C169、C170、C171、C172、C173、C178、C179、C183、C184、C186、C187、C180、C181、C182、C185、C188、C189、C211、C210、C212、C218、C220、C222、C223、C224、C227、C228、C221、C229、C240、C241、C248、C249、C250、C251、C252、C253、C254、C257、C258、C259、C480、C451、C481、C488、C482、C260、C261、C269、C300、C301、C310、C311、C312、C313、C318、C319、C320、C321、C322、C323、C328、C329、C340、C341、C342、C343、C348、C349、C384、C450、C380、C452、C381、C382、C388、C383、C390、C399、C410、C411、C412、C413、C400、C401、C414、C402、C403、C408、C409、C419、C470、C490、C471、C491、C472、C492、C473、C493、C474、C494、C475、C495、C476、C496、C478、C498、C479、C499、C430、D030、C431、D031、C432、D032、C433、D033、C434、D034、C435、D035、C436、D036、C437、D037、C438、D038、C439、D039、C440、C4A0、C441、C4A1、C442、C4A2、C443、C4A3、C444、C4A4、C445、C4A5、C446、C4A6、C447、C4A7、C448、C4A8、C449、C4A9、C500、C501、C502、C503、C504、C505、C506、C508、C509、C460、C461、C462、C464、C465、C463、C467、C469、C530、C531、C538、C539、C58、C541、C542、C543、C549、C540、C548、C561、C562、C569、C570、C571、C573、C572、C574、C510、C511、C512、C519、C518、C577、C578、C579、C620、C621、C629、C600、C601、C602、C609、C630、C631、C632、C608、C637、C638、C639、C670、C671、C672、C673、C674、C675、C676、C677、C678、C679、C641、C642、C649、C651、C652、C659、C661、C662、C669、C680、C681、C688、C689、C694、C696、C695、C690、C691、C692、C693、C698、C699、C710、C711、C712、C713、C714、C715、C716、C717、C719、C722、C723、C724、C725、C700、C709、C720、C721、C701、C729、E312、C740、C741、C749、C750、C751、C752、C753、C754、C755、C758、C759、C760、C761、C762、C763、C764、C765、C457、C768、C770、C7B0、C771、C772、C773、C774、C775、C778、C779、C780、C781、C782、J910、C783、C784、C785、C786、R180、C787、C788、C790、C791、C792、C793、C794、C795、C796、C797、C798、C799、C7A1、C7A8、C7B1、C7B8、C800、D3A8、C459、C801、C833、C846、C847、C852、C964、C965、C835、C837、C830、C838、C839、C865、C866、C817、C819、C810、C814、C811、C812、C813、C820、C821、C822、C823、C824、C825、C826、C828、C829、C840、C844、C849、C84A、C84Z、C860、C862、C863、C861、C841、C96A、C914、C960、C962、C831、C851、C858、C859、C864、C884、C969、C96Z、C900、C901、C882、C883、C888、C889、C902、C903、C910、C911、C91Z、C913、C915、C916、C91A、C919、C920、C924、C925、C926、C92A、C921、C922、C923、C92Z、C929、C933、C930、C931、C939、C93Z、C940、C942、C943、C948、C950 |
| **Psychiatric comorbidities** | *ICD-10-CM* codes |
| Depressive episodes | F329、F320、F321、F322、F323、F324、F325、F334、F339、F330、F331、F332、F333、F341 |
| Substance use-related disorder | F1123、F1193、F1323、F1393、F1423、F1523、F1593、F1720、F1721、F1722、F1729、F1923、F1993、F1115、F1125、F1195、F1215、F1225、F1295、F1315、F1325、F1395、F1415、F1425、F1495、F1515、F1525、F1595、F1120、F1121、F1320、F1321、F1420、F1421、F1220、F1221、F1520、F1521、F1620、F1621、F1820、F1920、F1821、F1921、F172、F121、F129、F161、F169、F131、F139、F111、F119、F141、F149、F151、F159、F191、F199、F181、F189、F550、F551、F552、F553、F554、F558 |
| Alcohol use-related disorder | F1012、F1022、F1023、F1092、F1026、F1096、F1097、F1027、F1015、F1025、F1095、F1014、F1018、F1024、F1028、F1098、F1019、F1029、F1094、F1099、F101、F102 |
| Neurocognitive disorder | F04、F09、F05、F015、F0390 |
| Sleep disorder | F519、F510、F511、F518、G472、F513、F514、F515、G478、G479、G470、G473、G471、G475、G476 |
| Anxiety disorder | F411、F419、F413、F418、F410、F419、F489、R452、R455、R456、F99 |
| Hyperactivity disorder | F909、F988、F901、F902、F908、F909、R418 |
| Personality disorder | F60、F34、F21、F68、F69、R4586 |
| Intellectual disabilities | F70、F71、F72、F73、F78、F79 |

Abbreviations: COPD = chronic obstructive pulmonary disease, HIV = human immunodeficiency virus, URI = upper respiratory tract infection

**e-Table 3.** Standardized mortality ratios of suicide mortality among patients with bipolar stratified by sex and age at baseline (cohort, N = 45211; mortality, n = 11246; suicide mortality, n = 1370)

|  | *N* | Follow-up Duration, years,  Mean (SD) | Number of Suicide Death Observed, *N* | Total Person-Years | Crude Incidence^a^ | Expected Number, *N* | SMR^b^ | 95% CI | P |
| --- | --- | --- | --- | --- | --- | --- | --- | --- | --- |
| Male | 21384 |  | 643 | 199735.2 | 321.9 | 40.5 | 15.9 | 14.7-17.2 | <.001 |
| <30 | 6008 | 11.6 (5.9) | 157 | 69746.8 | 225.1 | 4.9 | 32.2 | 27.4-37.7 | <.001 |
| 30-39 | 3562 | 10.0 (5.9) | 147 | 35520.7 | 413.8 | 8.5 | 17.4 | 14.7-18.4 | <.001 |
| 40-49 | 3876 | 9.6 (5.9) | 154 | 37234.7 | 413.6 | 9.8 | 15.7 | 13.3-18.4 | <.001 |
| 50-59 | 3360 | 8.7 (5.6) | 105 | 29221.5 | 359.3 | 8.1 | 12.9 | 10.6-15.6 | <.001 |
| ≧60 | 4578 | 6.1 (5.0) | 80 | 28011.4 | 285.6 | 9.2 | 8.7 | 6.9-10.8 | <.001 |
| Female | 23827 |  | 727 | 239001.7 | 304.2 | 25.2 | 28.8 | 26.8-31.0 | <.001 |
| <30 | 5800 | 10.4 (6.1) | 185 | 60134.3 | 307.6 | 2.4 | 76.8 | 66.1-88.7 | <.001 |
| 30-39 | 4665 | 11.5 (5.8) | 196 | 53737.6 | 364.7 | 6.0 | 32.9 | 28.4-37.8 | <.001 |
| 40-49 | 4509 | 11.1 (5.7) | 159 | 50106.5 | 317.3 | 6.0 | 26.3 | 22.4-30.7 | <.001 |
| 50-59 | 3806 | 10.1 (5.7) | 121 | 38281.3 | 316.1 | 4.6 | 26.6 | 22.1-31.8 | <.001 |
| ≧60 | 5047 | 7.3 (5.4) | 66 | 36742.1 | 179.6 | 6.2 | 10.6 | 8.2-13.5 | <.001 |
| Total | 45211 |  | 1370 | 438736.9 | 312.3 | 65.7 | 20.9 | 19.8-22.0 | <.001 |
| <30 | 11808 | 11.0 (6.0) | 342 | 129881.2 | 263.3 | 7.3 | 47.0 | 42.1-52.2 | <.001 |
| 30-39 | 8227 | 10.9 (5.9) | 343 | 89258.3 | 384.3 | 14.4 | 23.8 | 21.3-26.4 | <.001 |
| 40-49 | 8385 | 10.4 (5.8) | 313 | 87341.2 | 358.4 | 15.8 | 19.8 | 17.6-22.1 | <.001 |
| 50-59 | 7166 | 9.4 (5.7) | 226 | 67502.8 | 334.8 | 12.7 | 17.8 | 15.6-20.3 | <.001 |
| ≧60 | 9625 | 6.7 (5.2) | 146 | 64753.4 | 225.5 | 15.4 | 9.5 | 8.0-11.1 | <.001 |

^a^ Incidence rate: incident number/100,000 person-years

^b^ Standardized mortality ratio (SMR): observed number of cases with suicide mortality/expected number of cases; the expected suicide mortality was obtained by multiplying the cumulative contributed person-years of patients in the specified stratum of the cohort with schizophrenia by the incidence of suicide mortality (2000 to 2019) in the general population.

^c^ Indicates age at baseline; we calculated SMR on the basis of sex and period (year) adjustment.

**e-Table 4.** Univariable conditional logistic regression for calculating the adjusted risk ratios of psychiatric and physical comorbidities in a nested case–control study stratified by age (variables with P < 0.01 were included in the model).

| **Age group, years** | **<30** | **<30** |  | **30-39** | **30-39** |  | **40-49** | **40-49** |  | **50-59** | **50-59** |  | **>=60** | **>=60** |  |
| --- | --- | --- | --- | --- | --- | --- | --- | --- | --- | --- | --- | --- | --- | --- | --- |
|  | Cases | Controls | RR | Cases | Controls | RR | Cases | Controls | RR | Cases | Controls | RR | Cases | Controls | RR |
|  | N=  206 | N=  2060 |  | N=  302 | N=  3020 |  | N=  320 | N=  3200 |  | N=  290 | N=  2900 |  | N=  252 | N=  2520 |  |
| **Within 3 months before suicide mortality** | **%** | **%** |  | **%** | **%** |  | **%** | **%** |  | **%** | **%** |  | **%** | **%** |  |
| **Psychiatric comorbidities** |  |  |  |  |  |  |  |  |  |  |  |  |  |  |  |
| Depressive episode | 91 (44.2) | 353 (17.1) | 4.43** | 142 (47.0) | 727 (24.1) | 3.04** | 151 (47.2) | 884 (27.6) | 2.41** | 138 (47.6) | 870 (30.0) | 2.17** | 125 (49.6) | 609 (24.2) | 3.17** |
| Drug-induced mental disorder | 24 (11.7) | 66 (3.2) | 4.28** | 28 (9.3) | 121 (4.0) | 2.47** | 20 (6.3) | 97 (3.0) | 2.16* | 17 (5.9) | 71 (2.5) | 2.52** | 8 (3.2) | 26 (1.0) | 3.11* |
| Alcohol-induced mental disorders | 13 (6.3) | 30 (1.5) | 4.91** | 26 (8.6) | 153 (5.1) | 1.84* | 33 (10.3) | 186 (5.8) | 1.94* | 17 (5.9) | 112 (3.9) | 1.57 | 8 (3.2) | 41 (1.6) | 2.05 |
| Organic mental disorder | 7 (3.4) | 26 (1.3) | 2.72 | 8 (2.7) | 79 (2.6) | 1.01 | 12 (3.8) | 100 (3.1) | 1.21 | 14 (4.8) | 99 (3.4) | 1.44 | 37 (14.7) | 356 (14.1) | 1.05 |
| Sleep disorder | 51 (24.8) | 305 (14.8) | 1.97** | 122 (40.4) | 597 (19.8) | 2.78** | 115 (35.9) | 733 (22.9) | 1.90** | 96 (33.1) | 698 (24.1) | 1.57** | 87 (34.5) | 575 (22.8) | 1.78** |
| Anxiety states | 46 (22.3) | 209 (10.2) | 2.65** | 85 (28.2) | 415 (13.7) | 2.49** | 56 (17.5) | 505 (15.8) | 1.14 | 71 (24.5) | 511 (17.6) | 1.52* | 63 (25.0) | 366 (14.5) | 1.98** |
| Hyperactivity disorder | 5 (2.4) | 30 (1.5) | 1.75 | 0 (0.0) | 13 (0.4) | 0.00 | 5 (1. 6) | 14 (0.4) | 3.58 | - | - | 10.03 | 1 (0.4) | 3 (0.1) | 3.34 |
| Personality disorder | 37 (18.0) | 132 (6.4) | 3.47** | 47 (15.6) | 186 (6.2) | 2.92** | 33 (10.3) | 163 (5.09) | 2.21** | 25 (8.6) | 155 (5.3) | 1.72 | 21 (8.3) | 95 (3.8) | 2.46** |
| Intellectual Disabilities | 1 (0.5) | 49 (2.4) | 0.20 | 0 (0.0) | 32 (1.1) | 0.00 | 3 (0.9) | 18 (0.6) | 1.67 | 1 (0.3) | 13 (0.5) | 0.77 | 0 (0.0) | 4 (0.2) | 0.00 |
| **Physical illnesses** |  |  |  |  |  |  |  |  |  |  |  |  |  |  |  |
| **Cardiovascular diseases** |  |  |  |  |  |  |  |  |  |  |  |  |  |  |  |
| Hypertension | 4 (1.9) | 28 (1.4) | 1.46 | 14 (4.6) | 125 (4.1) | 1.13 | 66 (5.8) | 264 (5.8) | 0.73 | 60 (20.7) | 670 (23.1) | 0.86 | 109 (43.3) | 1011 (40.1) | 1.14 |
| Ischemic Heart Disease | 1 (0.5) | 3 (0.2) | 3.34 | 7 (2.3) | 32 (1.1) | 2.21 | 17 (1.5) | 38 (0.8) | 0.33 | 11 (3.8) | 171 (5.9) | 0.63 | 33 (13.1) | 301 (11.9) | 1.11 |
| Other Forms Of Heart Disease | 12 (5.8) | 29 (1.4) | 4.31** | 22 (7.3) | 53 (1.8) | 4.46** | 66 (5.8) | 64 (1.4) | 1.49 | 24 (8.3) | 139 (4.8) | 1.80 | 37 (14.7) | 227 (9.0) | 1.74* |
| Congestive heart failure | - | - | 10.00 | 4 (1.3) | 7 (0.2) | 5.71* | 8 (0.7) | 12 (0.3) | 0.00 | 10 (3.45) | 44 (1.5) | 2.31 | 9 (3.57) | 121 (4.8) | 0.74 |
| **Respiratory diseases** |  |  |  |  |  |  |  |  |  |  |  |  |  |  |  |
| Pneumonia | 8 (3.9) | 34 (1.8) | 2.40 | 13 (4.3) | 51 (1.7) | 2.64* | 13 (4.1) | 64 (2.0) | 2.07 | 17 (5.9) | 62 (2.1) | 2.81** | 18 (7.1) | 118 (4.7) | 1.58 |
| COPD | 3 (1.5) | 15 (0.7) | 2.02 | 8 (2.7) | 39 (1.3) | 2.10 | 7 (2.2) | 84 (2.6) | 0.83 | 10 (3.5) | 127 (4.4) | 0.78 | 27 (10.7) | 237 (9.4) | 1.17 |
| Chronic bronchitis | 1 (0.5) | 7 (0.3) | 1.43 | 5 (1.7) | 23 (0.8) | 2.21 | 6 (1.9) | 49 (1.5) | 1.23 | 8 (2.8) | 81 (2.8) | 0.99 | 20 (7.9) | 150 (6.0) | 1.39 |
| Asthma | 4 (1.9) | 38 (1.8) | 1.06 | 7 (2.3) | 75 (2.5) | 0.93 | 10 (3.1) | 93 (2.9) | 1.08 | 9 (3.1) | 116 (4.0) | 0.77 | 15 (6.0) | 134 (5.3) | 1.13 |
| Upper respiratory tract infection | 10 (4.9) | 133 (6.5) | 0.74 | 19 (6.3) | 227 (7.5) | 0.82 | 17 (5.3) | 216 (6.8) | 0.77 | 11 (3.8) | 178 (6.1) | 0.60 | 19 (7.5) | 153 (6.07) | 1.26 |
| **Gastrointestinal diseases** |  |  |  |  |  |  |  |  |  |  |  |  |  |  |  |
| Chronic hepatic disease | 4 (1.9) | 27 (1.3) | 1.49 | 25 (8.3) | 157 (5.2) | 1.69 | 24 (7.5) | 227 (7.1) | 1.06 | 18 (6.2) | 266 (9.2) | 0.66 | 23 (9.1) | 168 (6. 7) | 1.42 |
| Ulcer disease | 9 (4.4) | 59 (2.9) | 1.57 | 23 (7.6) | 140 (4.6) | 1.71 | 27 (8.4) | 243 (7.6) | 1.12 | 37 (12.8) | 268 (9.2) | 1.44 | 47 (18.6) | 289 (11.5) | 1.77** |
|  |  |  |  |  |  |  |  |  |  |  |  |  |  |  |  |
| Cerebrovascular Diseases | 1 (0.5) | 14 (0.7) | 0.71 | 8 (2.65) | 35 (1.16) | 2.31 | 8 (2.5) | 72 (2.3) | 1.11 | 11 (3.79) | 135 (4.7) | 0.81 | 36 (14.3) | 335 (13.3) | 1.09 |
| Diabetes mellitus | 3 (1.5) | 28 (1.4) | 1.07 | 16 (5.3) | 109 (3.6) | 1.5 | 21 (6.6) | 279 (8.7) | 0.74 | 46 (15.9) | 517 (17.8) |  | 59 (23.4) | 634 (25.2) | 0.91 |
| Cancer | 1 (0.5) | 9 (0.4) | 1.11 | 2 (0.7) | 35 (1.2) | 0.57 | 12 (3.8) | 108 (3.4) | 1.12 | 21 (7.2) | 169 (5.8) | 1.26 | 22 (8.7) | 153 (6.1) | 1.48 |
| Connective tissue disease | 2 (1.0) | 18 (0.9) | 1.11 | 4 (1.3) | 27 (0.9) | 1.50 | 4 (1.3) | 58 (1.8) | 0.69 | 8 (2.8) | 55 (1.9) | 1.48 | 6 (2.4) | 58 (2.3) | 1.04 |
| Moderate or severe renal disease | - | - | 14.98* | 5 (1.7) | 14 (0.5) | 3.66 | 7 (2.2) | 33 (1.0) | 2.14 | 8 (2.8) | 64 (2.2) | 1.26 | 17 (6.75) | 125 (5.0) | 1.39 |
| HIV infection | 2 (1.0) | 8 (0.4) | 2.50 | 3 (1.0) | 19 (0.6) | 1.60 | 0 (0.0) | 8 (0.3) | 0.00 | - | - | 0.00 | - | - | - |
| Atopic dermatitis and related conditions | 1 (0.5) | 17 (0.8) | 0.59 | 3 (1.0) | 18 (0.6) | 1.67 | 4 (1.3) | 21 (0.7) | 1.92 | 2 (0.7) | 18 (0.6) | 1.11 | 1 (0.4) | 20 (0.8) | 0.50 |
| Irritable bowel syndrome | 2 (1.0) | 15 (1.0) | 1.33 | 6 (2.0) | 40 (1.3) | 1.50 | 9 (2.8) | 61 (1.9) | 1.50 | 9 (3.1) | 58 (2.0) | 1.57 | 14 (5.6) | 57 (2.3) | 2.53* |
| Hyperlipidemia | 2 (1.0) | 23 (1.1) | 0.87 | 22 (7.3) | 121 (4.0) | 1.87* | 21 (6.6) | 267 (8.3) | 0.77 | 33 (11.4) | 422 (14.6) | 0.75 | 38 (15.1) | 481 (19.1) | 0.75 |
|  |  |  |  |  |  |  |  |  |  |  |  |  |  |  |  |

*p<0.01, **p<0.001; RR: risk ratio

**e-Table 5.** Multivariable conditional logistic regression for calculating the adjusted risk ratios of psychiatric and physical comorbidities in a nested case–control study stratified by age (variables with P < 0.01 were included in the model).

| **Age group, years** | **<30** | **<30** |  | **30-39** | **30-39** |  | **40-49** | **40-49** |  | **50-59** | **50-59** |  | **>=60** | **>=60** |  |
| --- | --- | --- | --- | --- | --- | --- | --- | --- | --- | --- | --- | --- | --- | --- | --- |
|  | Cases | Controls | aRR | Cases | Controls | aRR | Cases | Controls | aRR | Cases | Controls | aRR | Cases | Controls | aRR |
|  | N=  206 | N=  2060 |  | N=  302 | N=  3020 |  | N=  320 | N=  3200 |  | N=  290 | N=  2900 |  | N=  252 | N=  2520 |  |
| **Within 3 months before suicide mortality** | **%** | **%** |  | **%** | **%** |  | **%** | **%** |  | **%** | **%** |  | **%** | **%** |  |
| **Psychiatric comorbidities** |  |  |  |  |  |  |  |  |  |  |  |  |  |  |  |
| Depressive episode | 91 (44.2) | 353 (17.1) | 3.62** | 142 (47.0) | 727 (24.1) | 2.16** | 151 (47.2) | 884 (27.6) | 2.22** | 138 (47.6) | 870 (30.0) | 2.11** | 125 (49.6) | 609 (24.2) | 2.98** |
| Drug-induced mental disorder | 24 (11.7) | 66 (3.2) | 2.88** | – | – | – | – | – | – | 17 (5.9) | 71 (2.5) | 2.09* | – | – | – |
| Alcohol-induced mental disorders | 13 (6.3) | 30 (1.5) | 3.10* | – | – | – | – | – | – | – | – | – | – | – | – |
| Sleep disorder | – | – | – | 122 (40.4) | 597 (19.8) | 2.17** | 115 (35.9) | 733 (22.9) | 1.64** | – | – | – | – | – | – |
| Anxiety states | – | – | – | – | – | – | – | – | – | – | – | – | 63 (25.0) | 366 (14.5) | 1.63* |
| Personality disorder | – | – | – | 47 (15.6) | 186 (6.2) | 1.79* | – | – | – | – | – | – | – | – | – |
|  |  |  |  |  |  |  |  |  |  |  |  |  |  |  |  |
| **Physical comorbidities** |  |  |  |  |  |  |  |  |  |  |  |  |  |  |  |
| Other forms of heart disease | 12 (5.8) | 29 (1.4) | 3.38* | 22 (7.3) | 53 (1.8) | 3.48** | – | – | – | – | – | – | – | – | – |
| Pneumonia | – | – | – | – | – | – | – | – | – | 17 (5.9) | 62 (2.1) | 2.64** | – | – | – |
|  |  |  |  |  |  |  |  |  |  |  |  |  |  |  |  |

*P < 0.01, **P < 0.001; aRR: adjusted risk ratio
